# ELMO1 Is Upregulated in AML CD34+ Stem/Progenitor Cells, Mediates Chemotaxis and Predicts Poor Prognosis in Normal Karyotype AML

**DOI:** 10.1371/journal.pone.0111568

**Published:** 2014-10-31

**Authors:** Marta E. Capala, Edo Vellenga, Jan Jacob Schuringa

**Affiliations:** Department of Experimental Hematology, Cancer Research Center Groningen (CRCG), University Medical Center Groningen, University of Groningen, Groningen, the Netherlands; French Blood Institute, France

## Abstract

Both normal as well leukemic hematopoietic stem cells critically depend on their microenvironment in the bone marrow for processes such as self-renewal, survival and differentiation, although the exact pathways that are involved remain poorly understood. We performed transcriptome analysis on primitive CD34^+^ acute myeloid leukemia (AML) cells (n = 46), their more differentiated CD34^−^ leukemic progeny, and normal CD34^+^ bone marrow cells (n = 31) and focused on differentially expressed genes involved in adhesion and migration. Thus, Engulfment and Motility protein 1 (ELMO1) was identified amongst the top 50 most differentially expressed genes. ELMO1 is a crucial link in the signaling cascade that leads to activation of RAC GTPases and cytoskeleton rearrangements. We confirmed increased ELMO1 expression at the mRNA and protein level in a panel of AML samples and showed that high ELMO1 expression is an independent negative prognostic factor in normal karyotype (NK) AML in three large independent patient cohorts. Downmodulation of ELMO1 in human CB CD34^+^ cells did not significantly alter expansion, progenitor frequency or differentiation in stromal co-cultures, but did result in a decreased frequency of stem cells in LTC-IC assays. In BCR-ABL-transduced human CB CD34^+^ cells depletion of ELMO1 resulted in a mild decrease in proliferation, but replating capacity of progenitors was severely impaired. Downregulation of ELMO1 in a panel of primary CD34^+^ AML cells also resulted in reduced long-term growth in stromal co-cultures in two out of three cases. Pharmacological inhibition of the ELMO1 downstream target RAC resulted in a severely impaired proliferation and survival of leukemic cells. Finally, ELMO1 depletion caused a marked decrease in SDF1-induced chemotaxis of leukemic cells. Taken together, these data show that inhibiting the ELMO1-RAC axis might be an alternative way to target leukemic cells.

## Introduction

Acute myeloid leukemia (AML) is a heterogeneous disease in which various molecular events lead to a block in differentiation along the myeloid lineage, resulting in an accumulation of immature cells termed leukemic blasts, as well as impaired normal hematopoiesis. The current classification of AML based on morphological, cytogenetic and molecular abnormalities does not cover the heterogeneity in response to treatment, especially in the intermediate risk group constituting the majority (60%) of AML cases [1], [2]. Therefore, new markers that would allow a more accurate stratification of patients are needed to better guide treatment options. In recent years, several gene expression profiling (GEP) studies have been performed in order to identify leukemia-specific gene expression patterns and select a gene, or more likely a panel of genes, that could be used to better classify patients within the existing subgroups [3]–[8]. However, most of these studies were performed on the total mononuclear fraction (MNC) of AML samples, which contains mostly leukemic blasts.

It has been shown that leukemic stem cell (LSC) activity, similarly to normal hematopoietic stem cell (HSC) activity, is contained within the CD34^+^ fraction of AML cells in the vast majority of cases [9]–[13]. LSCs are defined as the cells able to transplant leukemia into immunodeficient recipients. In patients, LSCs are thought to be responsible for the relapse of disease and treatment failure [9], [14]–[16]. Therefore, we compared gene expression profiles of CD34^+^ AML, their CD34^−^ progeny and normal bone marrow (NBM) CD34^+^ cells [17], [18]. Here, we focused specifically on genes that might be involved in adhesion and/or migration properties and thus were able to identify Engulfment and Motility protein 1 (ELMO1) amongst the top 50 CD34^+^ AML-specific genes. ELMO1 is known to be a crucial link in the signaling cascade leading to the activation of Rac GTPases [19]–[21]. We identified ELMO1 as an independent prognostic marker in the normal karyotype (NK) AML subgroup and showed that high expression of ELMO1 was associated with poor prognosis in three independent cohorts of patients. Knockdown of ELMO1 or inhibition one of its downstream protein RAC impaired long-term expansion of leukemic cells on stroma, and ELMO1 depletion decreased the migration potential of hematopoietic cells towards an SDF-1 gradient.

## Materials and Methods

### Primary cell isolation and culture conditions

Neonatal cord blood (CB) was obtained from healthy full-term pregnancies after informed consent in accordance with the Declaration of Helsinki from the obstetrics departments of the University Medical Centre Groningen (UMCG) and Martini Hospital Groningen, Groningen, The Netherlands. All protocols were approved by the Medical Ethical Committee of the UMCG. After separation of mononuclear cells with lymphocyte separation medium (PAA Laboratories, Coble, Germany), CD34^+^ cells were isolated using a magnetically activated cell sorting (MACS) CD34 progenitor kit (Miltenyi Biotech, Amsterdam, The Netherlands). For liquid cultures, CD34^+^ cells were expanded in human progenitor growth medium (HPGM; Cambrex, Verviers, Belgium) supplemented with 100 ng/ml stem cell factor (SCF), FLT3 Ligand (Flt3L; both from Amgen, Thousand Oaks, USA) and thrombopoietin (TPO; Kirin, Tokyo, Japan). For the MS5 co-culture experiments, cells were grown in Gartner's medium consisting of α-modified essential medium (α–MEM; Fisher Scientific Europe, Emergo, The Netherlands) supplemented with 12.5% heat-inactivated fetal calf serum (Lonza, Leusden, The Netherlands), 12.5% heat-inactivated horse serum (Invitrogen, Breda, The Netherlands), 1% penicillin and streptomycin, 2 mM glutamine (all from PAA Laboratories), 57.2 µM β-mercaptoethanol (Merck Sharp & Dohme BV, Haarlem, The Netherlands) and 1 µM hydrocortisone (Sigma-Aldrich Chemie B.V., Zwijndrecht, The Netherlands). AML blasts from peripheral blood cells or bone marrow cells from untreated patients with AML were obtained and studied after informed consent in accordance with the Declaration of Helsinki, and the protocol was approved by the Medical Ethical Committee. AML mononuclear cells were isolated by density gradient centrifugation, and CD34^+^ cells were stained using CD34-PE antibody (BD Biosciences, San Jose, CA, USA) and selected by sorting on a MoFLo (DakoCytomation, Carpinteria, CA, USA). AML co-cultures were expanded in Gartner's medium supplemented with 20 ng/mL interleukin 3 (IL-3; Gist-Brocades, Delft, The Netherlands), granulocyte-colony stimulating factor (G-CSF; Rhone-Poulenc Rorer, Amstelveen, The Netherlands) and TPO.

### Cell lines and culture conditions

293T embryonic kidney cells (ACC-635 DSMZ, Braunschweig, Germany) and PG13 packaging cells (ATCC CRL-10686, Wesel, Germany) were grown in DMEM medium with 200 mM glutamine (BioWhittaker, Veries, Belgium) supplemented with 10% FSC and 1% penicillin and streptomycin. K562 myelogenous leukemia cells (ACC-10, DSMZ), TF-1 erythroleukemic cells (ACC-334, DSMZ) and THP-1 acute monocytic leukemia cells (ACC-16, DSMZ) were grown in RPMI medium with 200 mM glutamine (BioWhittaker) supplemented with 10% FCS, and 1% penicillin and streptomycin, and for TF-1 cells with 5 ng/ml granulocyte-macrophage colony stimulating factor (GM-CSF; Genetics Institute, Cambridge, MA, USA). MS-5 murine stromal cells (ACC-441, DSMZ) were grown in αMEM with 200 mM glutamine (BioWhittaker) supplemented with 10% FCS and 1% penicillin and streptomycin.

### Immunoblotting

Western blot analysis was performed according to standard protocols and as described previously [22]. Briefly, 5×10^5^ cells were lysed to prepare whole cell extract by boiling in Laemmli buffer for 5 min prior to separation on 12.5% SDS gels. After overnight transfer, membranes were blocked in phosphate-buffer saline (PBS) with 5% nonfat milk prior to incubating with antibodies. Binding of antibodies was detected by chemiluminescence, according to the manufacturer's instructions (Roche Diagnostics, Basel, Switzerland). Antibody against ELMO1 (ab2239) was obtained from Abcam (Abcam, Cambridge, UK) and was used at a dilution of 1∶1000. Antibody against phosphoPAK (#2601) was obtained from Cell Signaling (Cell Signaling, Leiden, The Netherlands) and used at a dilution of 1∶1000. Antibody against β-actin (#J2207, Santa Cruz Biotechnology, CA, USA) was used at a dilution of 1∶4000. Secondary antibodies were purchased from Dako Cytomation (Dako Cytomation, Glosturp, Denmark) and used at 1∶2500 dilutions.

### RNA extraction and Real-time PCT analysis

ELMO1 expression was assessed by quantitative real-time PCR (qRT-PCR) as described previously [23]. Briefly, total RNA was isolated using an RNeasy kit (Qiagen, Venlo, The Netherlands) following the manufacturer's recommendations. After reverse transcription using M-MuLV reverse transcriptase (Fermentas, St Leon-Roth, Germany), according to manufacturer's instructions, aliquots of cDNA were real-time amplified using iQ SYBR Green mix (Bio-Rad, CA, USA) on a MyIQ thermocycler (Bio-Rad). ELMO1 primers (forward primer: CCGGATTGTGCTTGAGAACA, reverse primer: CTCACTAGGCAACTCGCCCA) were obtained from Invitrogen. Expression was quantified using MyIQ software (Bio-Rad) and RPL27 expression was used to calculate relative expression levels of investigated genes according to the standard curve method. RPL27 was not differentially expressed in our AML versus NBM CD34^+^ cells (data not shown).

### Retro- and lentivirus generation and transduction

Stable PG13 producer cell lines of BCR-ABL retroviral constructs were generated and used as published previously [24]. Supernatants from the PG13 cells were harvested after 8–12 hours of incubation in HPGM before the retroviral transduction rounds and passed through 0.45-mm filters (Sigma-Aldrich). Before the first transduction round, CD34^+^ CB cells were pre-stimulated for 48 hours in HPMG supplemented with 100 ng/mL of SCF, Flt3L and TPO. Three rounds of transduction were performed on retronectin-coated 24-well plates in the presence of the same cytokines as for pre-stimulation and 4 µg/mlLpolybrene (Sigma-Aldrich). With the last round of transduction, lentiviral transduction with the constructs described below was performed.

Short hairpin RNA (shRNA) sequences targeting ELMO1 were derived from the literature [25], [26] and ligated into pHR'trip vector using AcsI and SbfI restriction sites. For the control, scrambled (shSCR) shRNA sequence was used. 293T embryonic kidney cells were transfected using FuGENE6 (Roche, Almere, The Netherlands) with 3 µg pCMV Δ8.91, 0,7 µg VSV-G, and 3 µg of vector constructs (pHR'trip-Scrambled shRNA [shSCR], or -ELMO1 shRNA [shELMO1]). After 24 hours, medium was changed to HPGM and after 12 hours, supernatant containing lentiviral particles was harvested and either stored at −80°C or used fresh for transduction of target cells. K562, TF-1, THP-1, isolated CD34^+^ CB cells that were pre-stimulated for 12 hours, or BCR-ABL-transduced CD34^+^ CB cells were subjected to 1 round of transduction with lentiviral particles in the presence of prestimulation cytokines and 4 µg/mL polybrene (Sigma) on retronectin-coated 24-well plates (Takara, Tokyo, Japan). After transduction, transduced green fluorescent protein-positive (GFP-positive), truncated nerve growth factor receptor-positive (NGFR-positive) or double-positive cells were sorted on a MoFlo sorter (Dako Cytomation). AML CD34^+^ cells were transduced as described previously [27]. Briefly, transductions were performed in 3 consecutive rounds of 8 to 12 hours with lentiviral supernatant supplemented with 10% FCS, IL-3, granulocyte-colony stimulating factor (G-CSF; Rhone-Poulenc Rorer, Amstelveen, The Netherlands) and TPO (20 ng/m each) and polybrene (4 µg/mL) on a retronectin-coated 24-wells plate. After washing away the virus supernatant, unsorted cells were used to initiate co-cultures.

### Liquid cultures, long-term cultures on stroma, CAFC and CFC assay

For liquid cultures, 3×10^5^ CD34^+^ CB cells or BCR-ABL cells were plated in 1 mL IMDM medium supplemented with 20% FSC, 1% P/S and 20 gn/mL of SCF and IL-3. 10^5^ CD34^+^ CB cells, 5×10^3^ BCR-ABL cells and 4×10^4^ cells (AML sample 3), 7×10^4^ cells (AML sample 1) or 12×10^4^ cells (AML sample 2) were plated onto a T25 flask pre-coated with MS5 stromal cells (ACC-441, DSMZ) in 5 ml of Gartner's medium in duplicate. AML culture medium was supplemented with 20 ng/mL IL-3, G-CSF and TPO. Co-cultures were kept at 37°C and 5% CO_2_ and cells were demi-depopulated weekly for analysis. For the inhibition of Rac activity, NSC2766 (NSC; Calbiochem, VWR, Amsterdam, The Netherlands) was added to the co-culture medium to the final concentration of 20 µM, 40 µM or 100 µM. CFC assays were performed as previously described [28]. 1000 CD34^+^ CB or 500 BCR-ABL-transduced cells were plated in 1 mL of CFC assay medium consisting of MethoCult H4230 (StemCell Technologies, France), 1% penicillin and streptomycin, 19% IMDM (PAA Laboratories), IL-3, interleukin-6 (IL-6; Gist-Brocades), G-CSF, SCF (all 20 ng/mL) and 1 U/mL erythropoietin (EPO; Cilag; Eprex, Brussels, Belgium) in duplicate directly after transduction and 10^4^ cells were used at later time points. After 14 days of culturing colony-forming unit granulocyte-macrophage (CFU-GM) and burst-forming unit erythroid (BFU-E) were scored. For CFC replate, colony cells were harvest after 14 days of culture, and 10^5^ cells were plated in 1 ml fresh CFC assay medium in duplicate. For CAFC assay, CFC assay medium was added to the co-cultures after 5 weeks. CAFC were counted 2 weeks after the addition of CFC medium by microscopic evaluation of co-cultures.

### Flow cytometry analysis and sorting

All fluorescence-activated cell sorter (FACS) analyses were performed on a FACScalibur (Becton-Dickinson [BD], Alpen a/d Rijn, the Netherlands) and data were analyzed using WinList 3D (Verity Software House, Topsham, USA). Cells were sorted on a MoFlo sorter. Antibodies: CD34-PE, NGFR-APC, CD14-PE, CD15-APC, CD71-APC and GPA-PE were obtained from BD. Viability was assessed using Annexin V APC (IQ Products, Groningen, The Netherlands) according to the manufacturer's recommendations. Briefly, cells were harvested, resuspended in 100 µL calcium buffer containing 5 µL Annexin V, and incubated for 20 min at 4°C in the dark, washed with 5 mL calcium buffer and binding of APC-conjugated Annexin V was measured by FACS.

### Migration assay

Migration assays were performed in transwell plates with 8 µm pore size (Corning Costar, Cambridge, UK) towards the gradient of SDF-1 (100 ng/mL; R&D Systems, Abingdon, UK). TF-1 or THP-1 leukemic cell lines were transduced with either shSCR or shELMO1, sorted and plated in the upper chamber of the transwell. Migration was allowed for 4 hours, after which the cells were harvested from the bottom chamber and counted by FACS using TruCOUNT counting beads (BD).

### Statistical analysis and transcriptome datasets used

Statistical analyses were performed with SPSS software (IBM, Amsterdam, The Netherlands), release 16.0. The association between the transcript level of ELMO1 and overall survival (OS) was tested in univariate Cox models. All values are expressed as means ± standard deviation (SD). Student's *t* test was used for all other comparisons. All tests were two tailed, and differences were considered statistically significant at *p*<0,05.

**Figure 1:**
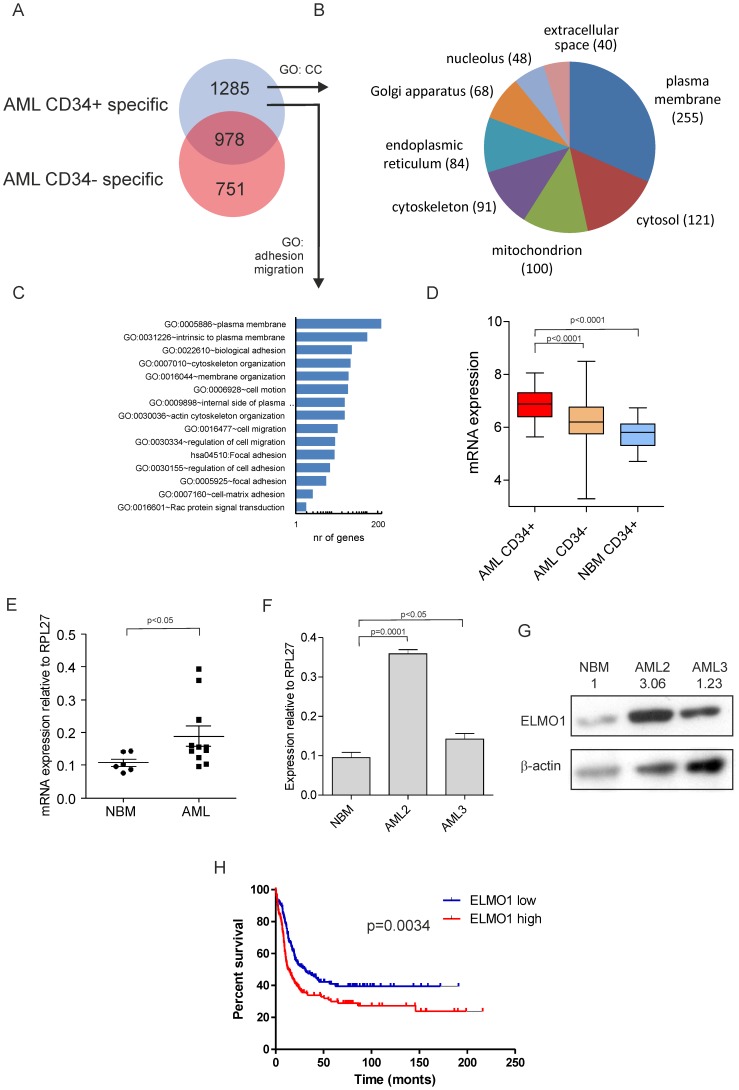
ELMO1 expression is increased in AML CD34^+^ cells and predicts poor prognosis in normal karyotype AML patients. (A) VENN diagram displaying the overlap in AML CD34^+^ specific and AML CD34− specific transcriptomes compared to NBM CD34^+^ cells [17], [18]. (B) 1677 AML CD34^+^-specific genes were subjected to Gene Ontology (GO) analysis for Cellular Component (CC) of which several CCs are shown. (C) 1677 AML CD34^+^-specific genes were subjected to GO analysis for terms associated with adhesion and migration. (D) ELMO1 mRNA was significantly higher expressed in AML CD34^+^ (n = 46) as compared to AML CD34^−^ cells (*p*<0.0001), as well as to NBM CD34^+^ cells (n = 31) (*p*<0.0001). (E) Increased expression of ELMO1 was further confirmed by independent Q-PCRs in a panel of 11 AML and 6 NBM samples. (F, G) The increase in ELMO1 mRNA was paralleled by increased protein levels in two representative cases. (H) High expression of ELMO1 predicts poor survival in a cohort of NK AML patients (based on [6], [29]) (p = 0.0034).

We made use of various previously published transcriptome datasets in our studies here. For Figure 1A,B, C and D we used data generated by ourselves containing AML CD34^+^ (n = 46) and NBM CD34^+^ (n = 31) [18] deposited in GEO under GEO30029). For Figure 1H we used data of intermediate risk AML samples (total cohort of 525 patients of which 300 were intermediate risk) [6], [29] deposited in GEO under GSE6891. At page 10 we describe data in two other NK AML datasets generated by the Bullinger lab indicating that high ELMO1 expression also predicts poor prognosis in these datasets and these data were already included in the Supplemental files of our previous paper [18]. Data for Figure S1B was derived from the HemaExplorer database (http://servers.binf.ku.dk/hemaexplorer/) [30]. For Figure S1C we used data from the Noverhstern lab [31].

## Results

### ELMO1 expression is increased in AML CD34^+^ cells and predicts poor prognosis in normal karyotype AML patients

Recently, we identified AML CD34^+^ leukemic stem cell-enriched transcriptomes by comparing gene expression profiles of paired AML CD34^+^ and CD34^−^ samples with those of normal BM CD34^+^ cells [17], [18]. Thus, 1677 AML CD34^+^-specific genes were identified (Figure 1A). Based on Gene Ontology (GO) analysis for Cellular Component (CC) this list of 1677 genes could be annotated to several CCs such as plasma membrane (253), cytosol (136), mitochondrion (116), cytoskeleton (108) and extracellular space (46) (Figure 1B). Since leukemic stem cells critically depend on their microenvironment in the bone marrow for processes such as self-renewal and survival, we focused on differentially expressed genes involved in adhesion and migration and an overview of selected GO terms is shown in Figure 1C. Thus, Engulfment and Motility protein 1 (ELMO1) was identified. In prior analyses we looked for prognostic significance by univariate cox regression analyses using the continuous transcript levels of the top 50 CD34^+^- specific genes and overall survival (OS) in a large series of de novo normal karyotype AML [18]. ELMO was present in this list, and could predict OS in 2 independent cohorts of patients (cohort 1: n = 163, p = 0.021, hazard ratio 1.782 and cohort 2: n = 218, p = 0.015, hazard ratio 1.657) [18]. ELMO1 is a crucial link in the signaling cascade that leads to activation of RAC GTPases and cytoskeleton rearrangements and therefore we decided to study its role in more detail. ELMO1 mRNA was significantly higher expressed in AML CD34^+^ (n = 46) as compared to AML CD34^−^ cells (*p*<0.0001), as well as to NBM CD34^+^ cells (n = 31) (*p*<0.0001) (Figure 1D). Increased expression of ELMO1 was further confirmed by independent Q-PCRs, which showed a good correlation with the Illumina BeadArrays data (Figure 1E and Figure S1A). Moreover, elevated mRNA expression was paralleled by an increase on the protein level in two representative cases (Figure 1F,G). Increased ELMO1 expression in AML compared to normal stem/progenitor cells was also observed in the HemaExplorer dataset [30] (Figure S1B). Finally, we analyzed the expression of ELMO1 in a third independent cohort of NK AML patients [6], [29], which again indicated that ELMO1 significantly predicts poor survival (p = 0.0034, Figure 1H).

### ELMO1 downmodulation in human CB CD34^+^ cells does not alter expansion, colony formation or differentiation, but results in a significant decrease in stem cell frequency

**Figure 2:**
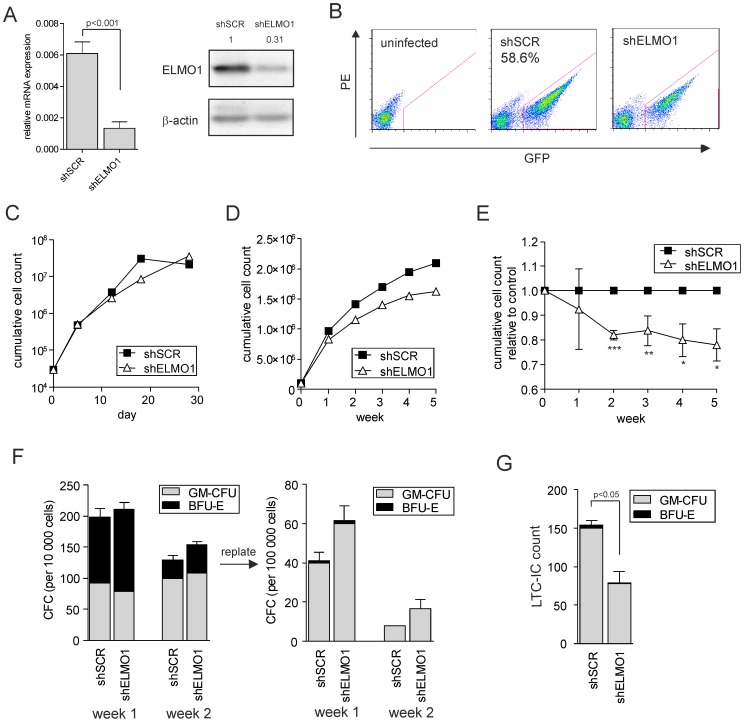
ELMO1 downmodulation in human CB CD34+ cells does not alter growth, colony formation or differentiation, but significantly decreases stem cell frequency. (A) K562 cells were transduced with control scrambled shRNA vector (shSCR) or with ELMO1-targeting shRNA vectors (shELMO1), sorted and used for RNA extraction. Quantitative PCR was performed to measure ELMO1 expression in transduced cells. ELMO1 mRNA levels were normalized against RPL27 mRNA expression. Alternatively, cells were used for Western blot analysis to determine ELMO1 protein levels. (B) FACS plots showing transduction efficiencies of cord blood (CB) CD34^+^ stem/progenitor cells transduced with shSCR or shELMO1. (C) 3×10^5^ transduced and sorted cells per group were plated in liquid culture and followed for thirty days. Cumulative cell count is showed representative of 3 independent experiments. (D) 10^5^ transduced and sorted cells per group were plated on MS5 stromal cells and kept in the co-culture for 5 weeks; cultures were demi-depopulated weekly for analysis. Weekly cumulative cell growth is shown for a representative experiment of 3 independent experiments and the average of those 3 experiments is shown in (E). (F) Suspension cells from MS5 co-cultures as described in panel D were analyzed for progenitor frequency by CFC assay. 10^4^ cells from each co-culture were plated in a CFC assay in methylcellulose in duplicate, and colonies were evaluated 2 weeks after plating. CFC cells were then harvested and 10^5^ cells were re-plated to form secondary CFCs. CFU-GM and BFU-E numbers are shown from a representative of 3 independent experiments; error bars indicate standard deviation. (G) LTC-IC frequencies were determined in bulk on MS5 stromal cells. After 5 weeks of co-culture methylcellulose was added to and colonies were scored two weeks later. CFU -GM, colony-forming unit-granulocyte-macrophage; BFU-E, burst forming unit-erythroid. * *P*<0.05, ** *P*<0.01, *** *P*<0.001.

In a recent study by Novershtern *et al.*, gene expression profiling was performed comparing 38 distinct purified populations of human hematopoietic cells [31]. Analysis of the data generated in this study revealed that ELMO1 is significantly more highly expressed in the most primitive hematopoietic compartment (HSCs) as compared with more differentiated cells (Figure S1C). Therefore, we set out to investigate the effect of ELMO1 depletion in CD34^+^ population of CB cells, enriched for hematopoietic stem and progenitor cells (HSPCs). In order to downregulate ELMO1 expression, lentiviral vectors containing ELMO1-targeting shRNA sequence were generated (shELMO1). The efficiency of downregulation was first tested in the K562 cell line, where transduction with shELMO1 significantly decreased ELMO1 expression both at the mRNA as well as protein level (Figure 2A). Subsequently, CB CD34^+^ cells were transduced with control (shSCR), or shELMO1-containing vectors, with transduction efficiencies of 59% for shSCR and 46% for shELMO1 (Figure 2B). Transduced cells were then sorted and plated either in liquid culture, or in a co-culture on stromal MS5 cells. The growth of CB CD34^+^ in liquid culture was followed for 28 days and within that time no significant differences were observed between the proliferation of ELMO1-depleted and control cells (Figure 2C). In contrast, the expansion of shELMO1-transduced cells during the 5-week co-culture on stroma was slightly lower than of the control cells (Figure 2D, E). We assessed cell differentiation along the myeloid lineage during the co-culture and saw that it was not affected by ELMO1 downregulation (Figure S2). Also the progenitor frequency and their self-renewal potential were not changed upon ELMO1 depletion. Of note, CFC cells from shELMO1-transduced group initiated slightly more colonies upon replate than the control, although this did not reach significance (Figure 2F). Finally, the LTC-IC frequency was assessed at the end of co-culture. In the shELMO1-transduced group significantly fewer colonies were observed (p = 0.042) indicative of the reduced stem cell frequency (Figure 2G). Overall, these data indicate that ELMO1 depletion did not significantly affect CB CD34^+^ proliferation in liquid culture and co-culture, myeloid differentiation or progenitor cell frequencies, but did cause a reduction of the most primitive stem cells.

### Depletion of ELMO1 results in a slight proliferative disadvantage and reduced replating capacity of BCR-ABL-transformed human CB CD34^+^

**Figure 3:**
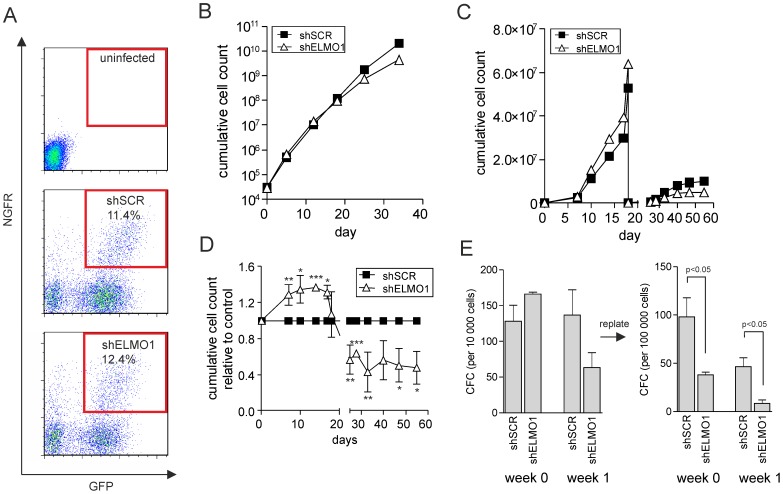
BCR-ABL-transformed human CB CD34^+^ show proliferative disadvantage and markedly reduced replating capacity upon ELMO1 depletion. (A) CB CD34^+^ stem/progenitor cells were double-transduced with BCR-ABL and either control scrambled shRNA vector (shSCR) or with ELMO1-targeting shRNA vector (shELMO1). FACS plots of transduction efficiency are shown. (B) 3×10^5^ double-transduced cells per group were plated in liquid culture and followed for 35 day. Cumulative cell count is shown representative of 3 independent experiments. (C) 5×10^3^ double-transduced cells were sorted per group and plated on MS5 stromal cells; cultures were demi-depopulated on indicated days for analysis and replated when stroma showed signs of detaching. Cumulative cell growth is shown for a representative experiment of 3 independent experiments and the average of those 3 experiments is shown in (D). (E) Suspension cells from MS5 co-cultures as described in panel B were analyzed for progenitor frequency by CFC assay. 10^3^ freshly transduced cells or 10^4^ cells from each co-culture were plated in a CFC assay in methylcellulose in duplicate, and colonies were evaluated 2 weeks after plating. CFC cells were harvested and 10^5^ cells were replated to assess secondary CFC formation. Total CFC numbers are shown from a representative of 3 independent experiments; error bars indicate standard deviation. * *P*<0.05, ** *P*<0.01, *** *P*<0.001.

Several studies have shown that RAC GTPases play an essential role in leukemic transformation mediated by BCR-ABL oncoprotein [32]–[37]. Since ELMO1 is involved in the activation of RAC proteins by the Dock180 family of GEFs, we hypothesized that depletion of ELMO1 would have a profound effect on the expansion of BCR-ABL-transformed cells. We performed a double transduction of CD34^+^ CB cells with BCR-ABL-containing retroviral vectors, together with shSCR or shELMO1 shRNA-containing lentiviral vectors. Double-transduced cells were then sorted (Figure 3A) and plated either in liquid culture or in a co-culture on MS5 stromal cells. Somewhat contrary to our expectations, proliferation of BCR-ABL cells was not markedly affected by ELMO1 downregulation during the 34 days of liquid culture (Figure 3B). Moreover, ELMO1-depleted BCR-ABL cells initially showed an increased proliferation in MS5 co-culture, but upon replating shELMO1-transduced cells did expand significantly less than the control suggesting that self-renewal properties were affected (Figure 3C, D). No marked differences were observed in the differentiation potential (data not shown). In the colony forming assay, shELMO1-transduced cells initiated the same number of colonies at week 1 of the co-culture, however at week 2 the number of colonies arising from shELMO1-transduced cells was lower that the control (Figure 3E). Moreover, significantly fewer secondary colonies were observed upon ELMO1 downregulation, again suggesting that self-renewal properties might be affected (Figure 3E). Taken together, these data indicate that ELMO1 depletion does not markedly affect BCR-ABL-transduced CB CD34^+^ cell proliferation in liquid culture and co-culture, but it decreases replating capacity of progenitor cells.

### Effects of ELMO1 depletion on long-term expansion of primary AML CD34^+^ cells on MS5 stroma

**Figure 4:**
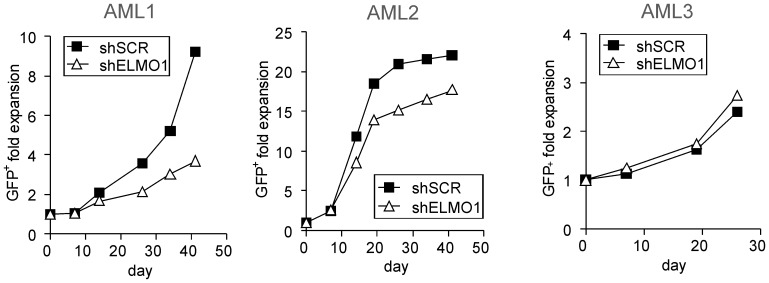
ELMO1 depletion in primary AML CD34^+^ cells impairs long-term expansion on stroma. AML CD34^+^ cells were transduced with control scrambled shRNA vector (shSCR) or with ELMO1-targeting shRNA vector (shELMO1). After washing away the virus all the cells were plated on MS5 stroma; cultures were demi-depopulated on indicated days for analysis. Cumulative cell growth is shown for each AML sample studied.

Next, we investigated the effect of ELMO1 downregulation in a panel of AML samples that showed high ELMO1 expression levels in the microarray profiling [18]. The following samples were used: 2003 022 (AML1), 2003 119 (AML2) and 2003 160 (AML3). The sample characteristics such as FAB classification, cytogenetic characteristics, risk group according to HOVON/SAKK protocols and FLT3/NPM mutation status are provided in Table 1. In order to downmodulate ELMO1, CD34^+^ cells were sorted from the AML mononuclear fraction and transduced with shSCR- or shELMO1-containing lentiviral vectors. Directly after transduction cells were plated on MS5 stroma and their expansion and GFP expression were followed during the co-culture. Transduction efficiencies obtained with shRNA constructs were variable, ranging from 16% in AML3 to 40% in AML1 and above 60% in AML2. AML3 ceased to expand beyond day 26 and within that time no significant differences in proliferation between shSCR- and shELMO1-transduced cells were observed. However, AML1 and AML2 could expand for as long as 41 days and in those cultures ELMO1-depleted cells grew markedly less than the control cells (Figure 4).

**Table 1:** Summary of clinical parameters of AML patients used in this study.

	2003 022	2003 119	2003 160
FAB classification	M5	M0	M1
Cytogenetic characteristics	NK	NK	complex karyotype
Risk group	intermediate	poor	poor
FLT3/NPM1	ITD/NPMc+	ITD/wt	wt/wt

Abbreviations: *FLT3*, fms-related tyrosine kinase 3; ITD, internal tandem duplication; NK, normal karyotype; *NPM1*, nucleophosmin 1; NPMc^+^, cytoplasmic dislocalization of NPM1; wt, wild type.

### Global inhibition of RAC activity severely impairs proliferation and survival of leukemic cells while ELMO1 depletion affects mostly cell migration

**Figure 5:**
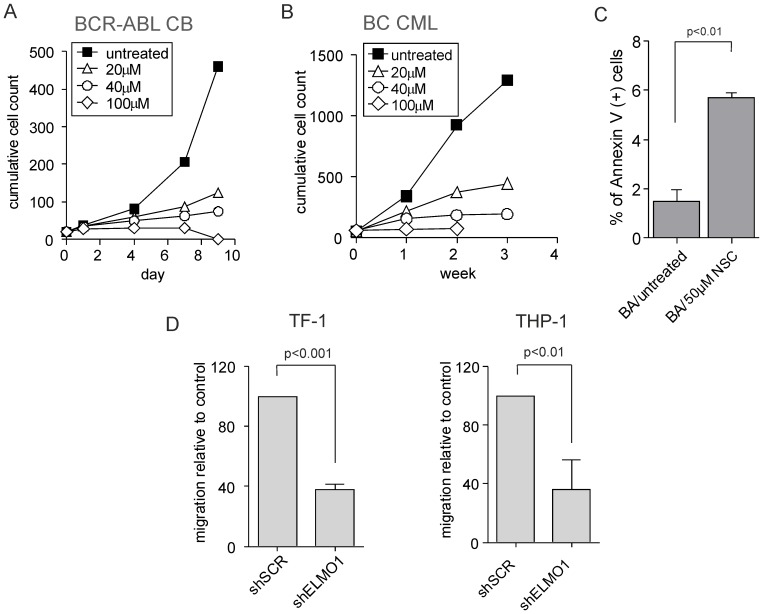
Global inhibition of RAC activity severely impairs proliferation and survival of leukemic cells while ELMO1 depletion strongly affects migration. (A) CB CD34^+^ cells were transduced with BCR-ABL-expressing vector, sorted and plated on MS5 stroma. Cells were allowed to proliferate for 5 days after which RAC inhibitor was added to the following concentrations: 20 µM, 40 µM or 100 µM. Co-cultures were demi-depopulated on indicated days for analysis. Cumulative cell count is shown representative of 3 independent experiments. (B) CD34^+^ cells were sorted from BC CML patient sample and plated on MS5 stroma. Cells were allowed to proliferate for 5 days after which RAC inhibitor was added to the following concentrations: 20 µM, 40 µM or 100 µM. Co-cultures were demi-depopulated on indicated days for analysis. Cumulative cell count is shown representative of 3 independent experiments. (C) BCR-ABL transduced cells as described in (A) were treated with 50 µM NSC for 3 days after which suspension cells were harvested and stained with Annexin V to assess apoptosis. (D) TF-1 and THP-1 cells were transduced with control scrambled shRNA vector (shSCR) or with ELMO1-targeting shRNA vector (shELMO1) and migration was evaluated in a transwell assay. The percentage of migrating cells relative to control is shown as an average of 3 independent experiments.

Subsequently, we investigated whether global inhibition of RAC activity, as a downstream target of ELMO1, would have an effect on the expansion of BCR-ABL-expressing leukemic cells. To this end, we used either BCR-ABL-transduced CD34^+^ CB cells, or primary blast crisis chronic myeloid leukemia (BC CML) cells. After plating on stroma, cells were allowed to expand and form cobblestones, after which the RAC inhibitor NSC23766 (NSC) was added to the co-cultures in concentrations of 20 µM to 100 µM. In BCR-ABL CB cells, inhibition of RAC activity caused a marked decrease in cell proliferation, apparent as early as 4 days after the addition of NSC. By day 9, cultures treated with 20 µM and 40 µM of NSC ceased to expand, while cells treated with 100 µM of inhibitor were depleted from the co-culture (Figure 5A). Analysis of phosphorylated PAK as a readout for RAC activity revealed that 40 µM of NSC23766 was sufficient to almost completely abolish RAC activity in the treated cells (Figure S3A). NSC-treated co-cultures showed an increased level of apoptotic cells, and the cells did not recover even after discontinuation of the treatment (Figure 5C and Figure S3B). Similarly, primary BC CML cells were highly sensitive to RAC inhibition. Co-cultures treated with the highest concentration of NSC did not survive past week 2, while cells treated with 20 µM or 40 µM of the inhibitor stopped expanding at were lost from the co-culture at week 3 (Figure 5B). The data are directly in line with our previously published data on the effects of the RAC inhibitor NSC on primary AML cells, where we also observed a strong reduction in long-term expansion on MS5 stromal cocultures [22].

Since global inhibition of RAC activity resulted in a much more severe phenotype than downregulation of ELMO1, we hypothesized that the activation via ELMO1-Dock180 pathway may be important for only some functions of RAC proteins. RAC GTPases have a well-described role in regulating migration and chemotaxis [38]; therefore we investigated the affected of ELMO1 depletion on the migratory potential of leukemic cells. To this end we transduced leukemic cell lines TF-1 and THP-1 with either shSCR- or shELMO1-containing lentiviral constructs. Depletion of ELMO1 resulted in an approximately 20% decrease of phospho-PAK activity and a slight proliferative disadvantage (Figure S4A, S4B). However, when we performed a transwell assay on either shSCR- or shELMO1-transduced leukemic cell lines we observed a marked decrease in the percentage of cells migrating towards the SDF-1 gradient upon ELMO1 downmodulation, indicative of a reduced chemotaxis (Figure 5D). Interestingly, treatment with RAC inhibitor resulted in a very pronounced apoptotic response in those cell lines, with virtually all cells dead within 3 days of treatment with 100 µM of NSC (Figure S4C, S4D). Therefore, while abolishing RAC activity was detrimental to the survival and proliferation of leukemic cells, inhibiting ELMO1-Dock180 activation pathway seemed to affect mostly their migratory potential.

## Discussion

The current study shows that ELMO1 is more highly expressed in AML CD34^+^ cells as compared with NBM CD34^+^ cells, and that this high expression has a prognostic significance in the NK subset of AML patients. Depletion of ELMO1 modestly impaired expansion in the long-term cultures of oncogene-transduced or primary leukemic CD34^+^ cells, while chemotaxis towards an SDF-1 gradient was significantly reduced.

Analysis of the data generated in the study by Novershtern et al. revealed that ELMO1 was significantly higher expressed in the most primitive hematopoietic compartment (HSCs) as compared with the more differentiated cells [31]. Importantly, in a recent proteome analysis of the plasma membrane fractions of CB CD34^+^ cells, we have found ELMO1 to be specifically associated with the plasma membrane of CD34^+^/CD38^−^ HSCs (data not shown). Taken together, these results identified ELMO1 as a protein most abundant in the primitive compartment of the hematopoietic system and suggested that ELMO1 might contribute to the biology of HSCs. Although downregulation of ELMO1 in CB CD34^+^ cells did not significantly affect proliferation, differentiation or progenitor cell frequencies, depletion of ELMO1 did result in a significant decrease of stem cell frequencies as determined by LTC-IC assay. Considering that increased expression of ELMO1 was predominantly observed within the most primitive stem and progenitor cells it is conceivable that those cells are most affected by downregulation of ELMO1.

We identified ELMO1 to be an AML34^+^-specific gene in a recent transcriptome analysis of paired AML CD34^+^ and CD34^−^ samples, and BM CD34^+^ cells [17], [18]. Although to date there is no data available on the involvement of ELMO1 in the hematopoietic malignancies, RAC proteins have been identified as crucial factors in the BCR-ABL- and MLL-AF9-induced leukemic transformation [32]–[37]. This led us to hypothesize that the increased expression of ELMO1 that we observed in leukemic cells may act by increasing RAC activity in these cells. ELMO1 downregulation in BCR-ABL-transduced CB CD34^+^ cells or in primary AML CD34^+^ cells initially did not strongly affect their long-term growth in stromal co-cultures, but upon serial replating of MS5 cocultures or upon serial replating of CFCs a significant reduction was observed. In two out of three AML cases a significant reduction in long-term growth on MS5 assays was also observed. Nevertheless, the effect of global RAC inhibition in BCR-ABL-expressing cells was far more pronounced than that of ELMO1 downregulation, both on the RAC activity levels and the cell proliferation and survival. These data are directly in line with our previously published data in primary AML cells, where we also observed a strong reduction in long-term expansion on MS5 stromal cocultures [22]. One possible explanation for differences in sensitivity towards ELMO or RAC knockdown might be that the residual ELMO1 expression in the transduced cells was still sufficient to maintain RAC activity and function. Also, it is plausible that redundancy exists between other members of the ELMO family that would compensate for ELMO1 loss, or alternatively other pathways might have become dominant in activating RAC upon ELMO1 depletion. Further studies are required to resolve these issues.

Previous studies have shown that increased expression of ELMO1 was responsible for the malignant behavior of ovarian and breast cancer, as well as glioma cells [25], [39]–[41]. In those cases, ELMO1 played a role in conferring the migratory and invasive properties of the malignant cells. Accordingly, when we assessed migratory potential of leukemic cell lines, we saw that it was reduced upon ELMO1 downmodulation. It is therefore possible that an increased expression of ELMO1 in LSCs confers migratory properties that contribute to the malignant phenotype and worse overall survival of AML patients.

Overall, we have shown that ELMO1 is more highly expressed in primitive leukemic cells and that it predicts prognosis in NK AML patients. Further studies using *in vivo* leukemia models are necessary to assess whether ELMO1 depletion could affect LSC homing and long-term engraftment, and ultimately whether targeting ELMO1 might have therapeutic benefit in the treatment of leukemia patients.

## Supporting Information

Figure S1 (A) Relative mRNA expression of ELMO1 was analyzed by Q-PCR in a panel of 11 AML samples and the correlation with Illumina BeadsArray expression data was assessed. Calculated r2 = 0.6. (B) Relative mRNA expression of ELMO1 (derived from HemaExplorer) was analyzed across different AML subtypes and compared to NBM populations. Number of samples per group: AML1-ETO n = 39; APL n = 37; inv16/t16 n = 28; t(11q23) n = 38; HSC_BM n = 7; HPC_BM n = 4. (C) Comparison of mRNA expression of ELMO1 in various human hematopoietic populations analyzed in the Novershtern dataset.
(TIF)

Figure S2 **CD34^+^ CB cells were transduced with either control shSCR or shELMO1 constructs, and 105 transduced and sorted cells per group were plated on MS5 stromal cells and kept in the co-culture for 5 weeks.** Cultures were demi-depopulated weekly and suspension cells were analyzed for differentiation along myeloid lineages. Percentage of CD14/CD15-double negative, CD14-positive and CD15-positive cells are shown.
(TIF)

Figure S3 (A) CB CD34^+^ cells were transduced with BCR-ABL-expressing vector, sorted and plated on MS5 stroma. Cells were allowed to proliferate for 5 days after which RAC inhibitor NSC was added to the following concentrations: 20 µM, 40 µM or 100 µM. After 3 days of treatment suspension cells were collected and phospho-PAK levels were assessed by Western blot. Quantification of phospho-PAK levels relative to control is indicated above each lane. (B) BCR-ABL-expressing cells as 3 described in (A) were treated with 50 µM NSC and co-cultures were demi-depopulated on indicated days for analysis. After 20 days NSC was washed away from the culture and treated cells were culture for additional 13 days after which all the cells were harvested for analysis. Cell counts are shown representative of 3 independent experiments.
(TIF)

Figure S4 (A) THP-1 cells were transduced with either control shSCR or shELMO1 vector and sorted. After 5 days of culture expression of phospo-PAK in transduced cells was analyzed by Western blot. Quantification of phospho-PAK levels relative to control is indicated above each lane. (B) shSCR- or shELMO1-transduced THP-1 cells were cultured for 9 days and cells were counted on the indicated time points. Cumulative cell count is shown representative of 3 independent experiments. (C) THP-1 cells were treated with either 50 µM or 100 µM NSC for 3 days and then stained with Annexin V to assess apoptosis. FACS plots representative of 3 independent experiments are shown and quantification of Annexin V (+) cells is shown in (D).
(TIF)
