# Supplementary figures and images for: ELMO1 Is Upregulated in AML CD34+ Stem/Progenitor Cells, Mediates Chemotaxis and Predicts Poor Prognosis in Normal Karyotype AML
Source: PLoS One. 2014 Oct 31;9(10):e111568. doi: 10.1371/journal.pone.0111568 (PMC4216115; doi:10.1371/journal.pone.0111568)

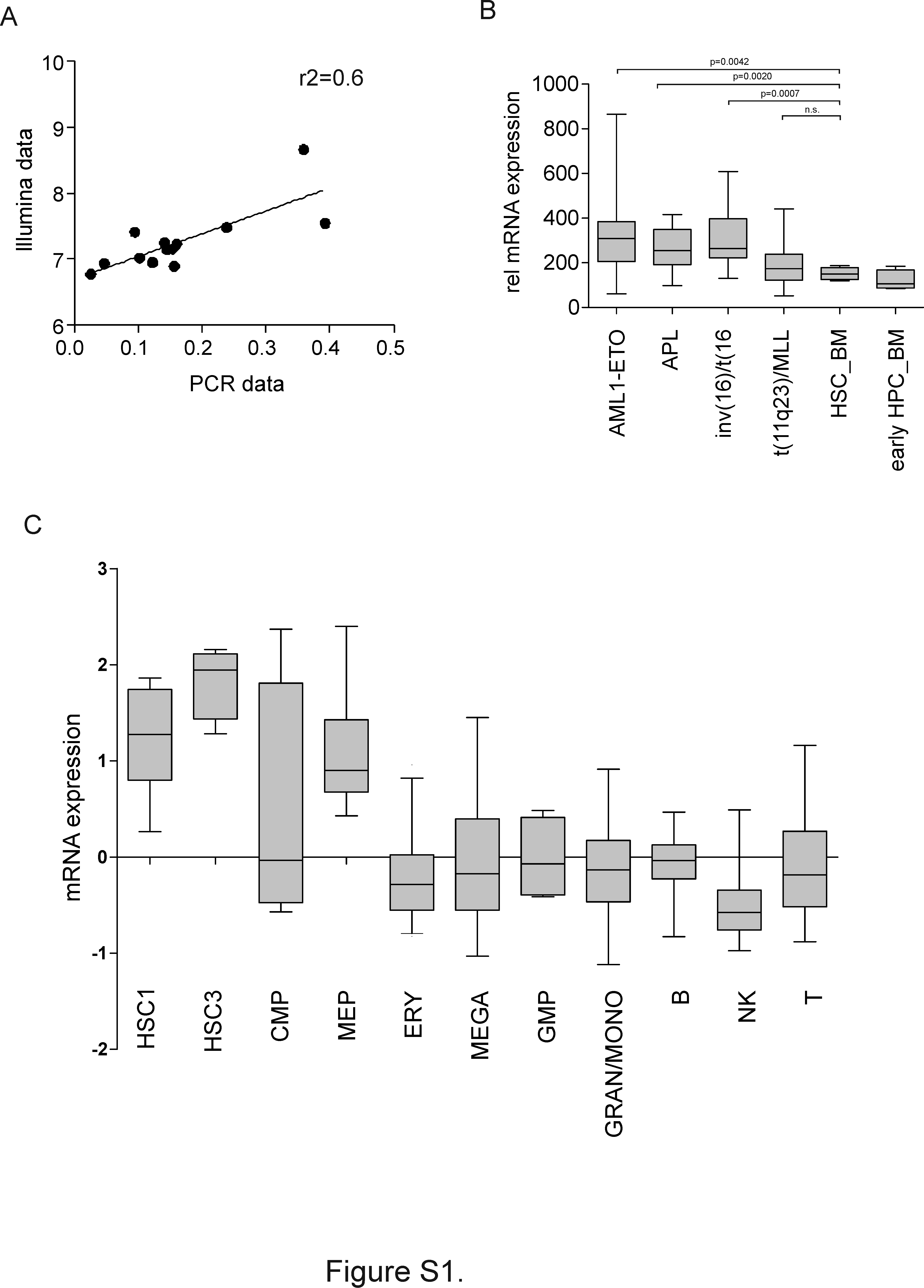

Supplement: Figure S1 — (A) Relative mRNA expression of ELMO1 was analyzed by Q-PCR in a panel of 11 AML samples and the correlation with Illumina BeadsArray expression data was assessed. Calculated r2 = 0.6. (B) Relative mRNA expression of ELMO1 (derived from HemaExplorer) was analyzed across different AML subtypes and compared to NBM populations. Number of samples per group: AML1-ETO n = 39; APL n = 37; inv16/t16 n = 28; t(11q23) n = 38; HSC_BM n = 7; HPC_BM n = 4. (C) Comparison of mRNA expression of ELMO1 in various human hematopoietic populations analyzed in the Novershtern dataset. (TIF) [file pone.0111568.s001.tif]

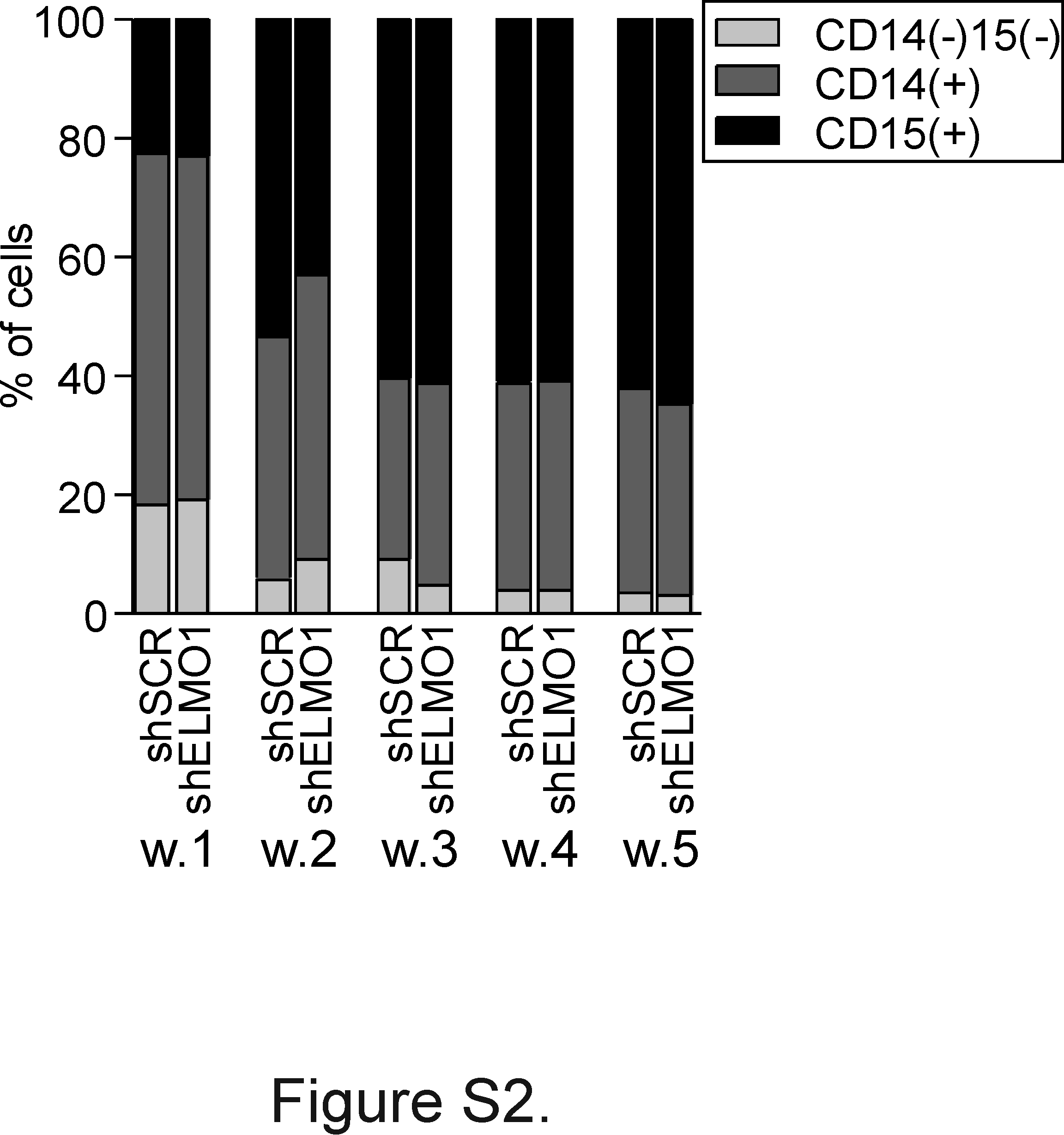

Supplement: Figure S2 — CD34+ CB cells were transduced with either control shSCR or shELMO1 constructs, and 105 transduced and sorted cells per group were plated on MS5 stromal cells and kept in the co-culture for 5 weeks. Cultures were demi-depopulated weekly and suspension cells were analyzed for differentiation along myeloid lineages. Percentage of CD14/CD15-double negative, CD14-positive and CD15-positive cells are shown. (TIF) [file pone.0111568.s002.tif]

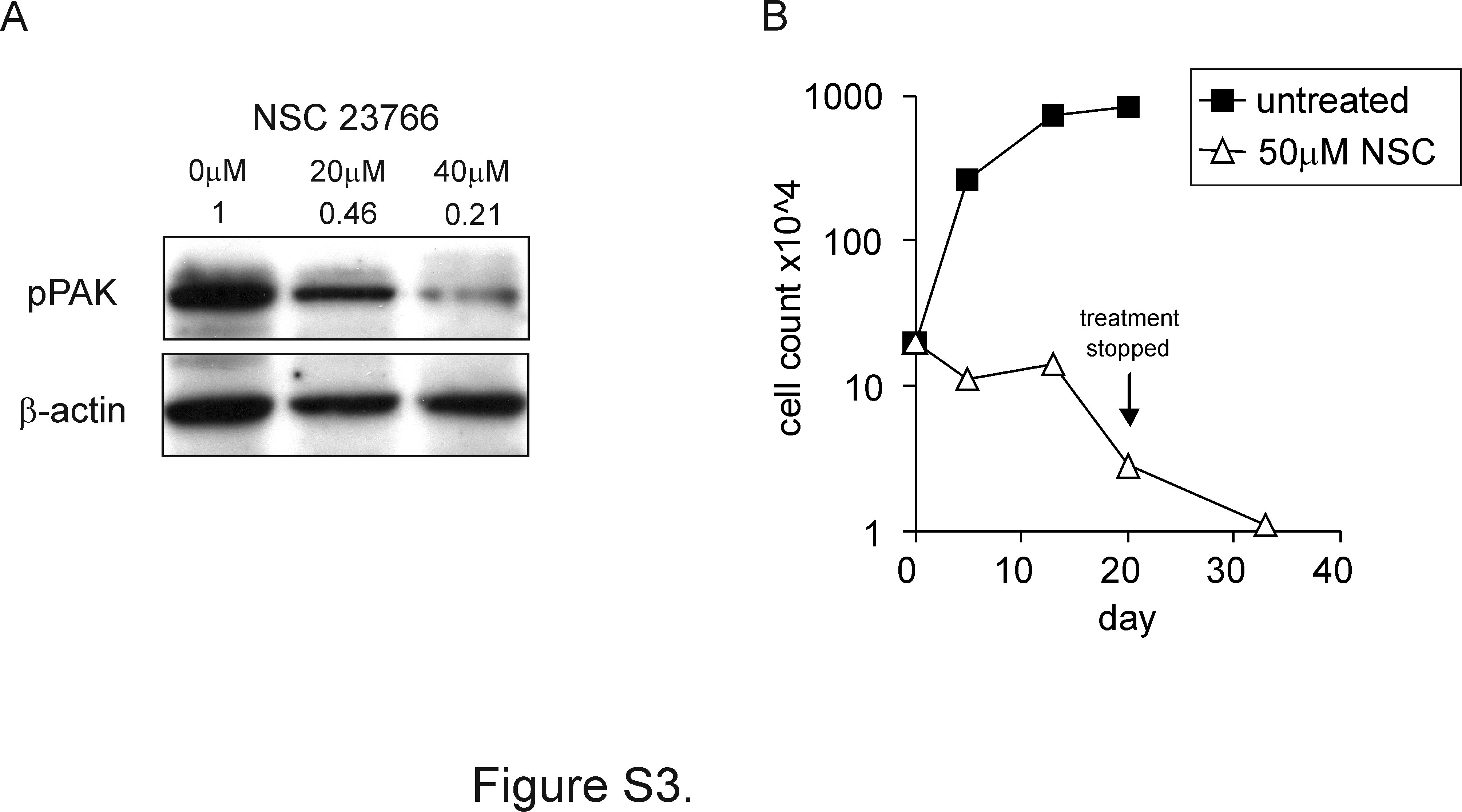

Supplement: Figure S3 — (A) CB CD34+ cells were transduced with BCR-ABL-expressing vector, sorted and plated on MS5 stroma. Cells were allowed to proliferate for 5 days after which RAC inhibitor NSC was added to the following concentrations: 20 µM, 40 µM or 100 µM. After 3 days of treatment suspension cells were collected and phospho-PAK levels were assessed by Western blot. Quantification of phospho-PAK levels relative to control is indicated above each lane. (B) BCR-ABL-expressing cells as 3 described in (A) were treated with 50 µM NSC and co-cultures were demi-depopulated on indicated days for analysis. After 20 days NSC was washed away from the culture and treated cells were culture for additional 13 days after which all the cells were harvested for analysis. Cell counts are shown representative of 3 independent experiments. (TIF) [file pone.0111568.s003.tif]

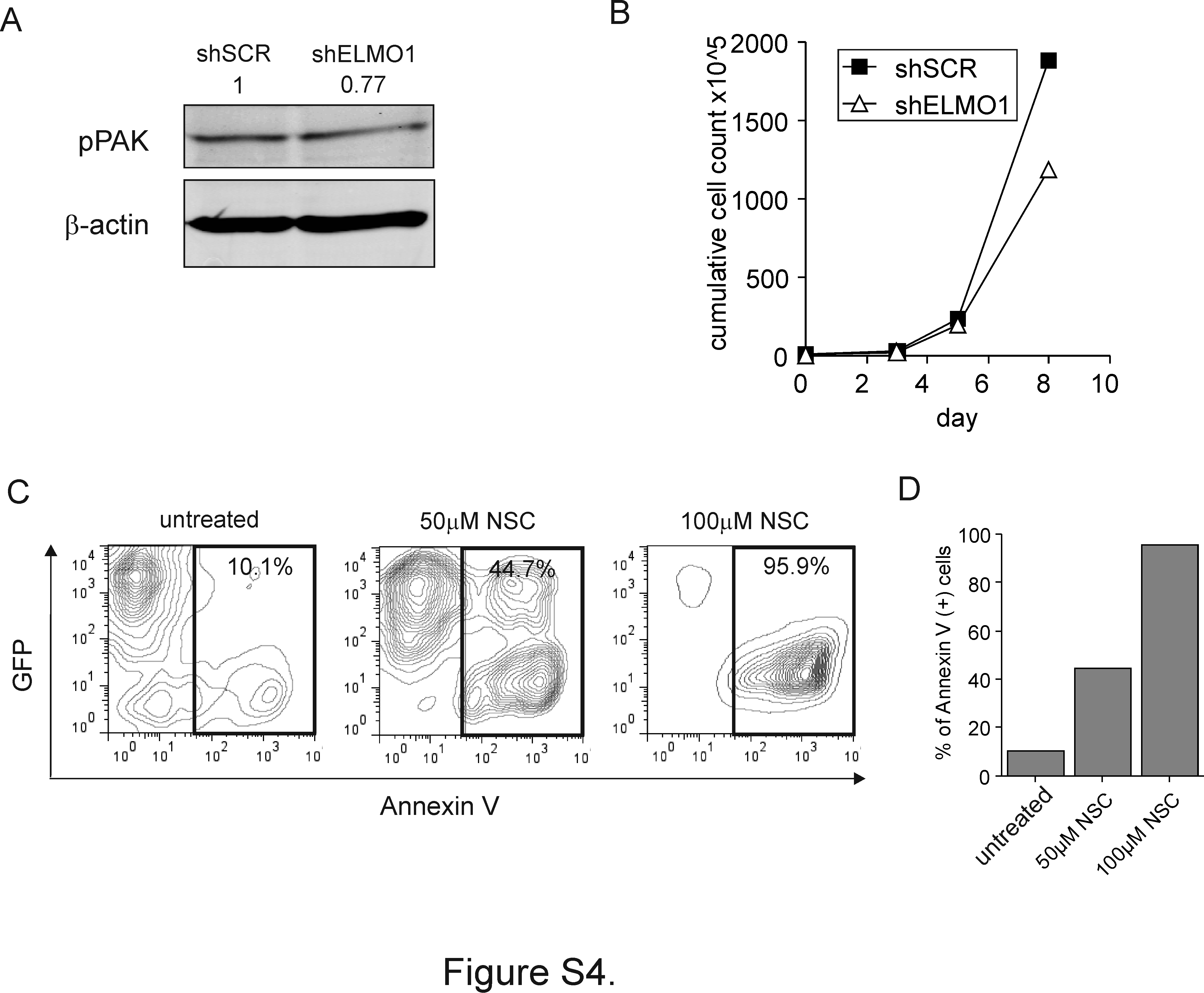

Supplement: Figure S4 — (A) THP-1 cells were transduced with either control shSCR or shELMO1 vector and sorted. After 5 days of culture expression of phospo-PAK in transduced cells was analyzed by Western blot. Quantification of phospho-PAK levels relative to control is indicated above each lane. (B) shSCR- or shELMO1-transduced THP-1 cells were cultured for 9 days and cells were counted on the indicated time points. Cumulative cell count is shown representative of 3 independent experiments. (C) THP-1 cells were treated with either 50 µM or 100 µM NSC for 3 days and then stained with Annexin V to assess apoptosis. FACS plots representative of 3 independent experiments are shown and quantification of Annexin V (+) cells is shown in (D). (TIF) [file pone.0111568.s004.tif]
